# American Pharmaceutical Association

**Published:** 1867-06

**Authors:** Frederick Stearns

**Affiliations:** Prest. Am. Phar. Ass’n.; Detroit


### *American Pharmaceutical Association.*

Notice is hereby given that the fifteenth (15th) annual meeting of the American Pharmaceutical Association will be held in New York City, commencing at 3 o'clock P.M., on the second Tuesday in September (10th), 1867.

A suitable room has been procured by the local Secretary in the University Buildings, on University Place, corner of Waverly Place.

Aside from the importance of the reports to be submitted, it may be of interest to the Association to know that several of our members, now abroad, will act as delegates of the Association to the International Congress of Pharmaceutists, at Paris, August 21st, and will return in time to be present at the session in New York.

A cordial invitation is extended to all engaged in trade or manufactures connected with pharmacy, to send specimens of their stock or products, for exhibition during the session. These may be sent to P. W. Bedford, Secretary American Pharmaceutical Association, New York City, notifications to that effect being addressed to him, in advance, by mail, to 709 Sixth Avenue.

FREDERICK STEARNS,
*Detroit, May 1st, 1867.* *Prest. Am. Phar. Ass'n.*

